# Biological Stoichiometry in Human Cancer

**DOI:** 10.1371/journal.pone.0001028

**Published:** 2007-10-10

**Authors:** James J. Elser, Marcia M. Kyle, Marilyn S. Smith, John D. Nagy

**Affiliations:** 1 School of Life Sciences, Arizona State University, Tempe, Arizona, United States of America; 2 Department of Microbiology, Molecular Genetics, and Immunology, University of Kansas Medical Center, Kansas City, Kansas, United States of America; 3 Department of Biology, Scottsdale Community College, Scottsdale, Arizona, United States of America; University of Utah, United States of America

## Abstract

**Background:**

A growing tumor in the body can be considered a complex ecological and evolutionary system. A new eco-evolutionary hypothesis (the “Growth Rate Hypothesis”, GRH) proposes that tumors have elevated phosphorus (P) demands due to increased allocation to P-rich nucleic acids, especially ribosomal RNA, to meet the protein synthesis demands of accelerated proliferation.

**Methodology/Principal Findings:**

We determined the elemental (C, N, P) and nucleic acid contents of paired malignant and normal tissues from colon, lung, liver, or kidney for 121 patients. Consistent with the GRH, lung and colon tumors were significantly higher (by approximately two-fold) in P content (fraction of dry weight) and RNA content and lower in nitrogen (N):P ratio than paired normal tissue, and P in RNA contributed a significantly larger fraction of total biomass P in malignant relative to normal tissues. Furthermore, patient-specific differences for %P between malignant and normal tissues were positively correlated with such differences for %RNA, both for the overall data and within three of the four organ sites. However, significant differences in %P and %RNA between malignant and normal tissues were not seen in liver and kidney and, overall, RNA contributed only ∼11% of total tissue P content.

**Conclusions/Significance:**

Data for lung and colon tumors provide support for the GRH in human cancer. The two-fold amplification of P content in colon and lung tumors may set the stage for potential P-limitation of their proliferation, as such differences often do for rapidly growing biota in ecosystems. However, data for kidney and liver do not support the GRH. To account for these conflicting observations, we suggest that local environments in some organs select for neoplastic cells bearing mutations increasing cell division rate (“r-selected,” as in colon and lung) while conditions elsewhere may select for reduced mortality rate (“K-selected,” as in liver and kidney).

## Introduction

Despite a greatly expanded knowledge base, post-occurrence cancer survival rates have shown only modest improvements in recent decades [1]. Thus, new approaches are needed that can integrate the diverse body of knowledge in this field to yield a better understanding of cancer and improve available therapies. One increasingly important emphasis in cancer biology is to consider the neoplasm and host as a complex ecological system in which genetically heterogeneous tumor populations undergo evolutionary change [2],[3]. This emphasis becomes increasingly compelling in light of findings of “cryptic cancer”, in which modern molecular screening indicates the widespread presence of cells containing known oncogene-specific mutations in otherwise healthy tissue [4]. While such “precancers” may still lack key mutations for complete oncogenic transformation, such observations also suggest that important aspects of a genetically divergent cell's environment may be critical in its eventual development into a physiologically significant tumor. However, eco-evolutionary approaches are not yet widespread and few attempts have been made to operationalize the ecological mechanisms at play in a tumor-host ecosystem.

Ecological stoichiometry is the study of the balance of energy and multiple chemical elements in ecological interactions [5]. More recently, this approach has been extended to a broader set of evolutionary and functional questions beyond ecology; this extended theory is biological stoichiometry [6]. In this context, it has recently been proposed that tumor cells present an example of a stoichiometric syndrome in which there are close positive associations among growth rate, biomass RNA content (fraction of dry mass), and biomass phosphorus (P) content [7]. These associations occur because rapidly proliferating cells generally increase their allocation to P-rich ribosomal RNA to meet the elevated protein synthesis demands of high growth rate. This “Growth Rate Hypothesis” (GRH hereafter) has received considerable support in recent studies involving diverse biota ranging from fruit flies to bacteria [8]. One corollary of this hypothesis is that, all else being equal, P-rich biota should be more frequently limited by environmental or dietary P supply [8]. Thus, the GRH predicts that tumors may be susceptible to *in vivo* P-limitation of growth [7]. We tested the GRH in the context of cancer biology by evaluating the nitrogen (N), P, and nucleic acid (RNA, DNA) contents of paired malignant and adjacent normal tissue biopsies originating from colon, liver, kidney, and lung.

## Methods

### Sample Analyses and Database

Biopsy samples were obtained via the Cooperative Human Tissue Network (CHTN) of the National Cancer Institute. Samples were obtained nearly exclusively from primary tumors originating in four organs (liver, kidney, colon/rectum, or lung). According to standard CHTN procedures, samples of tumor and of healthy adjacent tissues were obtained, with a portion examined by a pathologist for diagnosis and the remaining material snap-frozen in liquid nitrogen and held at −70°C until shipment on dry ice to ASU where they were held at −80°C until further processing. For nucleic acid analysis, sub-samples from each biopsy sample were fractured on dry ice and 50–100 mg samples were immediately homogenized with 1 ml Trizol (Invitrogen™). Following established extraction procedures [9], after incubation for 10 min at room temperature one-fifth volume of chloroform was added and mixed, after which the phases were separated by centrifugation at 12,000 g for 15 min at 4°C. The organic phase was re-extracted, and the pooled aqueous phase was precipitated with isopropanol and centrifuged at 12,000 g for 10 min at 4°C. The pellet was then washed with cold 75% ethanol and re-centrifuged, as per the manufacturer's protocol. The final RNA product was treated with RNAse-free DNAse using DNA-free reagents (Ambion). DNA was extracted from frozen sub-samples using the QIAamp DNA minikit (QIAGEN™). Nucleic acid concentrations of extracts were then quantified using a NanoDrop® ND-1000 spectrophotometer. Since samples for nucleic acid analysis could not be dried for comparison with elemental analyses, for each organ we developed an empirical factor to convert fresh-frozen weight to dry weight. To assess possible RNA degradation during sample handling, all extracts were also subjected to a quantitative real-time PCR assay for amplification of the 177 bp mRNA of the hypoxanthine guanine phosphoribosyltransferase 1 (HPRT) housekeeping gene [10]. Samples indicating possible RNA degradation were excluded from analysis. Sub-samples for elemental analysis were dried and weighed and then analyzed for P by colorimetry [11] or for carbon (C) and N (via a PerkinElmer model 2400 Elemental Analyzer). All results were then expressed as a percentage of dry weight. To estimate the percentage of total biomass P contributed by RNA, RNA content was multiplied by 0.086, the mass fraction of RNA contributed by P [5], and then compared with total P content.

### Statistical Analysis

All comparative measures, i.e., %P, %N, N:P ratio, %RNA, %DNA and % of P contributed by RNA, were handled similarly. For each tissue of origin (colon/rectum, kidney, liver and lung), data were analyzed directly and after being summarized in pair-wise fashion for each patient as a ratio of malignant tissue to normal. Ratios for percentage of P contributed by RNA were log-transformed. Outliers, defined as any measurement falling more than 1.5 inner-quartile ranges beyond the nearest inner quartile for a specific tissue, were removed before further analyses. No more than one or two data points were removed as clear outliers in any given analysis. Deviations from normality were probed with D'Agostino's test for skewness and Anscombe-Glynn tests for kurtosis. We tested homogeneity of variance assumptions and also compared variances of normal and malignant tissues for all measures using the Fligner-Killeen test for homogeneity of variance, both for the overall data set and for each organ site. In every case but two, Fligner-Killeen agreed with an analogous Bartlett test. In both exceptions, the Bartlett algorithm was obviously affected by skewness in the data. If normality and variance assumptions were met, absolute data were analyzed by two-way analysis of variance (ANOVA) with organ (liver, kidney, colon, lung) and tissue type (normal, malignant) as independent variables. The pair-wise malignant/normal ratios were evaluated for significant differences between malignant and normal tissues for the four organ sites by using one-sample *t*-tests on the null hypothesis that all (four) parametric ratios equaled unity. If assumptions of normality were not met, we performed analogous one-sample Wilcoxon tests on the analogous null. Attained significance for these multiple comparisons was adjusted using Holm's procedure for each comparative measure. In addition, to evaluate among-site differences in chemical composition among healthy tissues, we also performed Kruskal-Wallis tests for each parameter. This test is robust against violations of the assumptions of ANOVA. We also report Pearson's product moment correlation coefficient between total P and RNA content. All statistical calculations were performed using R Statistical Software, version 2.1.1.

## Results and Discussion

**Figure 1:**
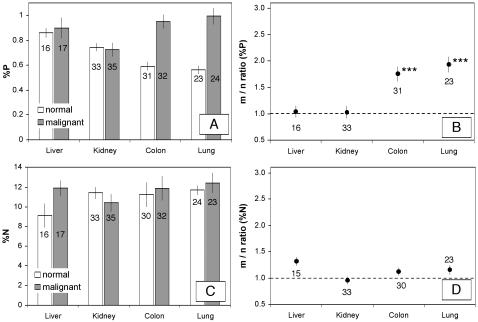
Absolute and relative values for elemental composition in normal and malignant tissues of liver, kidney, colon, and lung. A. and B. P content. C. and D. N content. The number of observations contributing to each mean is given by the number associated with each bar or point. Data in the right hand panels are expressed as the mean of the patient-specific ratios of malignant relative to normal tissue values (m/n ratio) for each parameter. The horizontal line shows an m/n ratio of one, indicating no difference between malignant and normal tissues. Error bars indicate±one standard error. Asterisks next to each symbol in the right hand panels indicate the results of the organ-specific *t*-test examining whether the m/n ratio differs from one for that organ (*** = p<0.0001; ** = 0.0001<p<0.001; * = 0.001<p<0.05; no asterisk = non-significant).

**Figure 2 pone-0001028-g002:**
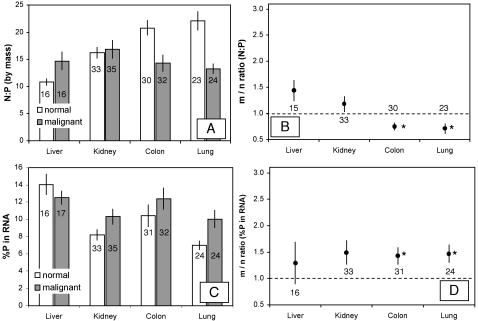
Absolute and relative (malignant/normal) values for N:P ratio and percentage of P in RNA for normal and malignant tissues of liver, kidney, colon, and lung. A. Absolute values and B. relative values for N:P ratio. C. Absolute values and D. relative values for %P in RNA. Data are expressed as in Figure 1.

**Figure 3 pone-0001028-g003:**
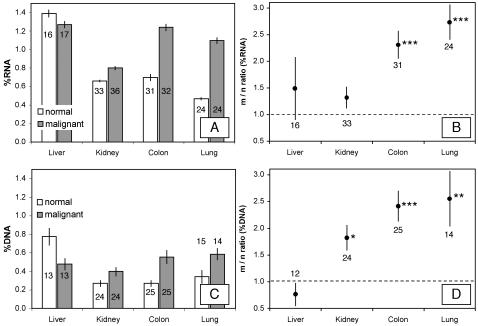
Absolute and relative (malignant/normal) values of biochemical parameters in normal and malignant tissues of liver, kidney, colon, and lung. A. and B. RNA content. C. and D. DNA content. Data are expressed as in Figure 1.

Analysis of variance indicated that the four organs sampled differed significantly overall in nearly all measures of elemental and biochemical composition (Table 1). P-content (percent of dry mass) was relatively high in normal samples (Figure 1A) from both kidney and liver (∼0.75–0.85%) compared to samples from colon and lung (∼0.55%), although this overall difference was only marginally significant (p = 0.06). In contrast, tissue N-content was significantly different among organs (p<0.015), primarily due to a somewhat decreased %N in liver (Figure 1C). N:P ratios in normal tissue inversely reflected those for P content (Figure 2A) and differed significantly among organs (p<0.015), reflecting lower N:P in kidney and especially liver. Among-organ differences in both RNA content (percent of dry mass) and DNA content for normal tissues (Figure 3A and 3C) were highly significant (p<10^−4^), being higher in liver (∼1.3% and 0.75% respectively) relative to normal samples from kidney, colon, and lung (0.5–0.7% and 0.25–0.35%, respectively). Finally, among-organ differences in %P in RNA for normal tissues also differed significantly (p<10^−4^). The percentage of total P contributed by P in RNA for normal tissues (Figure 2C) ranged from 7% (lung) to 14% (liver). As expected from the relatively uniform %C of major biomolecules [5], %C did not differ very much among organs, although the among-site differences were statistically significant (p<0.01). Median %C values for the four organs were: 48.9% (lung), 51.2 (colon), 51.2 (kidney), 51.4 (liver).

**Table 1:** Results of two-way Analysis of Variance (ANOVA) evaluating the statistical significance of differences in elemental and biochemical parameters in biopsy samples as a function of site (kidney, liver, colon, lung) and status (normal, malignant); the significance of the site-by-status interaction was also evaluated.

Source	d.f.	F	p<	K-W p<
%P				
site	3	2.47	0.06	10^−6^
status	1	34.1	10^−7^	
site×status	3	10.9	10^−5^	
residuals	203			
%N				
site	3	2.47	0.014	0.01
status	1	34.1	0.15	
site×status	3	10.9	0.0027	
residuals	202			
N∶P				
site	3	3.60	0.014	10^−6^
status	1	9.50	0.002	
site×status	3	7.5	10^−4^	
residuals	200			
%RNA				
site	3	13.37	10^−7^	10^−7^
status	1	25.7	10^−6^	
site×status	3	6.14	10^−3^	
residuals	205			
%DNA				
site	3	8.55	10^−4^	10^−4^
status	1	11.0	0.002	
site×status	3	9.12	10^−4^	
residuals	145			
%P in RNA				
site	3	7.36	10^−4^	10^−4^
status	1	5.85	0.016	
site×status	3	1.42	0.24	
residuals	203			

The final column reports the p value from the Kruskal-Wallis test for homogeneity of medians among normal tissues according to site (organ). This test is robust against violations of assumptions of normality and homogeneity of variances.

**Figure 4 pone-0001028-g004:**
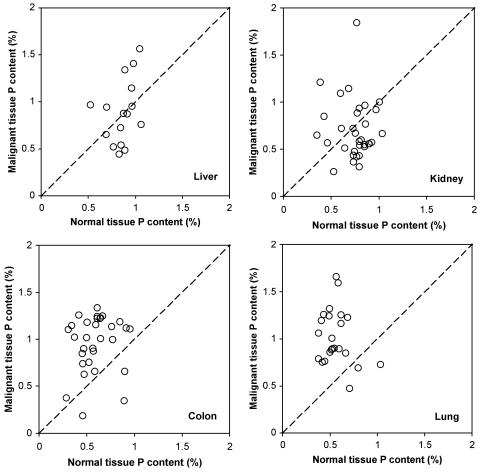
Scatter plots of patient-specific data for paired observations of P content (% of dry mass) for malignant (*y*-axis) and normal (*x*-axis) tissues in the four organs studied. The dotted line indicates the 1:1 relationship. A data point lying above the line indicates that P content is elevated in malignant tissues, relative to normal, in that patient.

**Figure 5:**
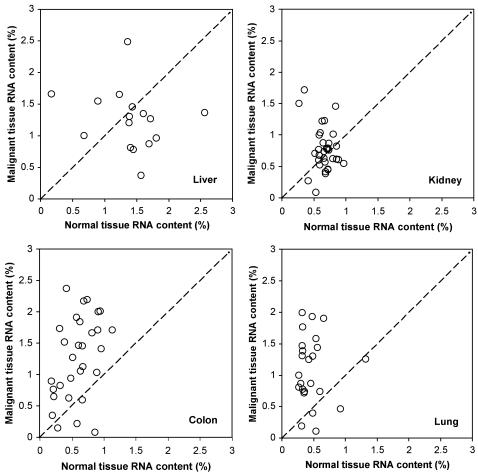
Scatter plots of patient-specific data for paired observations of RNA content (% of dry mass) for malignant (*y*-axis) and normal (*x*-axis) tissues in the four organs studied. The dotted line indicates the 1:1 relationship. A data point lying above the line indicates that RNA content is elevated in malignant tissues, relative to normal, in that patient.

Next we consider differences between malignant and normal tissues. There was generally greater variance in %P and %RNA in malignant tissues relative to normal tissues (see Figures 4 and 5), both for the overall data set and for each organ site considered separately (p<0.04 from Fligner-Killeen test, except for RNA content in liver where p = 0.80). These differences likely reflect the fact that malignant tissue samples contain variable mixtures of both normal and transformed cells while normal samples contain only normal cells. Consistent with the GRH, malignant tissues differed significantly from normal tissues in all parameters analyzed {except for %N (p = 0.15 in two-way ANOVA) and %C (p = 0.89); note that these are not predicted to differ under the GRH}. However, these differences depended on the organ from which the tumor samples were obtained (Figures 1–5, Table 1). These differences are more precisely assessed by considering the patient-specific data for each parameter in paired malignant and normal tissues (right hand panels in Figures 1–3 and scatter-plots for paired %P and %RNA data in Figures 4 and 5). In colon and lung, tumor P content was approximately double that in normal tissue (p<10^−3^, based on one-sample *t*-test; Figure 1B), while kidney and liver tumors did not differ (p>0.5) in P content from normal tissue. Since N-content was similar in malignant and normal tissues (except for liver, where N-content was somewhat higher in malignant samples; Figure 1D), N:P ratios (Figure 2B) were also significantly lower in colon and lung tumor samples (p<0.01) but did not differ in kidney and liver (p>0.08). Consistent with the GRH, there was a broad similarity between the patterns observed for P content and for RNA content (compare Figure 3B with Figure 1B and Figure 5 with Figure 4), as RNA concentrations in malignant tissue were ∼2.5-fold higher than in normal tissues for colon and lung (p<10^−4^) but not for kidney and liver (p>0.8). This pattern also held for DNA concentrations (p<0.02 for colon, lung, and kidney but p>0.4 for liver, Figure 3D), likely reflecting increased ploidy levels that are often observed in advanced tumors [12]. Finally, the percentage of P contributed by RNA was ∼1.5-times higher in malignant relative to normal tissues in all of the organs (Figure 2D), but this was significant only for colon and lung cancer (p<0.003; p>0.3 for liver and kidney). We also performed paired ANOVA for each parameter and obtained results highly consistent with the one-sample *t*-tests. Additional support for the GRH comes from comparison of patient-specific “residuals” from %P and %RNA data depicted in Figures 4 and 5. Finally, we determined the patient-specific differences between tumor and normal %P and between tumor and normal %RNA and evaluated, for the overall data set and within each organ site, the degree to which patients having large increases in %P in tumor tissue relative to normal tissue also had correspondingly large deviations in %RNA. There were significant positive correlations between these patient-specific differences both for the overall data set (p<10^−8^, *r* ^2^ = 0.28) and for three of the four organ sites (p<0.05, *r* ^2^ = 0.09 to 0.36; for colon, p = 0.18, *r* ^2^ = 0.02). This provides evidence that the GRH holds not only at the aggregate scale of the overall averages but also at the scale of individual patients.

While the findings just described are broadly consistent with the GRH, in our samples RNA contributed only ∼10% of total P, in contrast to a previous finding of ∼50% for bacteria, crustaceans, and insects [8]. The relatively low contribution of RNA to P in our samples may reflect methodological effects or real differences between these human tissues and the invertebrate and bacterial samples analyzed previously. For example, RNA degradation prior to sample freezing may result in an underestimate of the contribution of RNA to overall P. However, since we used analysis of the HPRT housekeeping gene to remove RNA samples that may have been significantly degraded, this is unlikely to have contributed significantly to our findings. Another possibility is that RNA extraction efficiency from our relatively large and biochemically heterogeneous tumor and normal samples was low compared to RNA recovery from small invertebrates and bacteria. If so, then a lower contribution of RNA to overall P would be observed. However, the relatively low value we observed for human tissues is likely to be realistic, as the contribution of RNA to biomass P is predicted to decline from humans to small invertebrates because growth rate and overall metabolic proliferation also decline with increasing body size [13].

Despite these issues, averaged total P content and RNA content for malignant or normal tissues for the four organ types showed a strong and significant positive relationship (p<0.001, *r* ^2^ = 0.72). In total, these results indicate that tumor development in lung and colon involves shifts of biochemical allocations, including both RNA and DNA, resulting in more than a two-fold increase in the mass-specific demand for phosphorus. We note that this increase is likely an underestimate of the elevated P demands of transformed cells, as the biopsy samples of tumor tissues likely involve an undetermined mixture of both transformed and normal cells along with acellular stromal matrix.

An obvious question emerging from our data is why tumor tissues are enriched in P and nucleic acids in colon and lung but not in liver and kidney. Long-standing theory of r/K-selection from evolutionary ecology [14] provides a hypothesis. In r/K selection theory, environmental conditions such as disturbance or high rates of predation are thought to favor individuals with rapid development rate and high fecundity, with a general trade-off in that they are weak competitors when resources become limiting [14]. This is “r selection.” (“r” refers to the maximum rate of population increase in population dynamic equations.) In contrast, “K selection” involves a scenario in which stable conditions result in an environment in which resources may often be insufficient to support maximal growth, thus favoring reduced mortality rates and improved competitive abilities. (“K” refers to the carrying capacity parameter in population equations.)

Applying these ideas to our data, we hypothesize that, because external epithelial tissues experience routinely unstable conditions imposing high levels of external mortality (for lung, especially under conditions of external stress, such as from cigarette smoke), tumors in lung and colon may reflect the outcome of long-term selection favoring cellular transformations increasing cell division rate (“r-selected”). In contrast, more stable local conditions in liver and kidney may instead predominantly favor transformed cells that have acquired lower rates of cellular mortality, such as reduced apoptosis (“K-selected”). Under the GRH, the r-strategy requires a particular biochemical allocation affecting biomass P content, but a K-strategy does not. These ideas can be tested by characterizing the particular genetic transformations that predominate in P-rich tumors vs. those that predominate in low-P tumors. Our hypothesis predicts that P-rich tumors should be dominated by cells with genetic lesions interfering with appropriate down-regulation of ribosome production or other cell-cycle checks while low-P tumors should be dominated by cells with mutations leading to inappropriately low rates of apoptosis or senescence.

Existing data hint at the potential validity of such a framework. For example, it is known that over-expression of c-*myc* protein leads to increased cellular proliferation via amplified rates of ribosome biogenesis [15] and that *myc* is over-expressed in 70% of colon cancers [16], consistent with the P-rich signature that we document (Figure 1). Thus, cellular transformation via *myc* represents a possible pathway to a P-rich “r-selected” tumor. We propose a similar scenario for tumorigenesis via mutations involving retinoblastoma protein, which is involved in regulation of RNA polymerases I and III [17]. With respect to development of “K-selected” tumors, possible genetic mechanisms may include loss-of-function mutations in *Fas*-mediated [18] or *COX*-mediated [19] signaling pathways to apoptosis. Indeed, *Fas*-mediated apoptosis provides an evolutionary mechanism by which tumor cells evade apoptosis signals via DcR3, a “decoy receptor” for the Fas protein ligand, FasL. DcR3 efficiently binds FasL and neutralizes its effectiveness as an effector molecule for cytotoxic T and NK cells. DcR3 has been shown to be significantly amplified in 50% of primary lung and colon tumors [20]. This evidence of selection for reduced apoptotic loss (a pathway consistent with K-selection, not r-selection) in lung and colon cancer is inconsistent with our argument that P-rich lung and colon tumors are the products of r selection. However, since many tumor suppressor genes and oncogenes have multiple direct and indirect effects on *both* cellular replication and cellular death (*p53* being a prominent example, [21]), it is likely that tumor development reflects the simultaneous operation of multiple mechanisms. It is also important to recognize that r/K selection theory proposes not a qualitative categorization of evolutionary outcomes but instead proposes a continuous gradient of relative contributions of r-selected and K-selected traits for any given species. This likely also holds for tumors. The challenge, amid the vast complexity of cancer genetics and gene expression, remains to identify which alternative pathways (replication acceleration vs. mortality reduction) predominate under which conditions and why. Emerging genomic and transcriptomic approaches hold considerable promise for functionally categorizing different tumors along an r/K continuum. For example, genomic analyses of high-P tumors may reveal that they are dominated by cells containing mutations associated primarily with cell-cycle regulation or ribosome biogenesis while low-P tumors may be dominated by cells harboring genetic changes resulting in reduced rates of apoptosis or cellular senescence.

Ecological studies have shown that P-rich organisms are generally more susceptible to P-limited growth due to insufficient supplies of P from the external environment or diet [5]. Whether neoplasms with amplified P content (such as the ∼2-fold increase of P-content in colon and lung tumors) also experience P-limited growth remains to be tested. However, existing clinical data do suggest that the elevated P demands of proliferating tumors can have body-wide physiological effects. For example, oncological hypophosphatemia has been hypothesized in some cases to reflect transfer of serum PO_4_ into replicating tumor cells [22]. Tumor-induced osteomalacia [23], a relatively rare condition in which tumor cells release (newly identified) phosphatonins that lead to elevated renal PO_4_ loss and mobilization of PO_4_ from bones, is another intriguing example of a connection between tumor development and P homeostasis. While preliminary, our findings indicate that, for at least some tumors, the requirements for the key nutrient element phosphorus differ substantially from those of normal tissues. Future studies are needed to evaluate whether the amplification of P content in tumor tissues that we document has physiological significance and whether it may provide additional avenues for therapy. Additional work further characterizing the stoichiometric signature of tumor tissues and examining the dynamical consequences of observed differences for tumor progression and for selection among clonal lineages is needed as well.
